# Preliminary pharmacological activity of the methanolic extract of *Premna integrifolia* barks in rats

**Published:** 2014

**Authors:** Hajera Khatun, Rajib Majumder, Al Mamun, Efte Kharul Alam, Safkath Ibne Jami, Badrul Alam

**Affiliations:** 1 *Department of Pharmacy, Southeast University, Dhaka, Bangladesh*; 2 *Department of Pharmacy, Atish Dipankar University of Science and Technology, Dhaka, Bangladesh*; 3 *Department of Pharmacy, Rajshahi University, Rajshahi, Bangladesh*; 4 *Department of Pharmacy, University of Asia Pacific, Dhaka, Bangladesh*; 5 *Department of Pharmacy, Jahangirnagar University, Savar, Dhaka, Bangladesh*

**Keywords:** *Analgesic*, *Antibacterial*, *CNS depressant*, *Inflammation*, *Premna integrifolia*

## Abstract

**Objective:** *Premna integrifolia *Linn (Family: Verbenaceae) synonym of *Premna serratifolia* has tremendous medicinal value. Preliminary pharmacological studies were performed on the methanolic extract of *Premna integrifolia *(MEPI) bark to investigate neuropharmacological, analgesic, and anti-inflammatory activities.

**Materials and methods:** Neuropharmacology study was done by open field and hole cross test whereas acetic acid writhing test and formalin induced pain was done for analgesic activity of MEPI. Carrageenan induced inflammatory model was considered for anti-inflammatory activity evaluation.

**Results:** A statistically significant (p0.05) decrease in locomotor activity was observed at all doses in the open-field and hole-cross tests. The extract significantly (p0.05) and dose dependently reduced the writhing reflex in the acetic acid-induced writhing test as well as licking response in the formalin induced inflammatory pain. At 200 mg/kg body weight dose, MEPI showed 71.16% inhibition in carrageenan induced anti-inflammatory activity.

**Conclusion:** The finding of this study suggests that MEPI will provide scientific support for the use of this species in traditional medicine.

## Introduction

The use of the medicinal plants is increasing in many countries and 35% of drugs contain natural products. Expensive and prolong uses of synthetic drugs demonstrated more side and toxic effects

that make efforts to introduce new plant derived cheaper drugs, having fewer or no side effects. During this process, the investigation of the efficacy of plant-based drugs used in the traditional medicine has been paid great attention because they are 

cheap and little side effects (Rakh and Chaudhari, 2010 ▶). In Bangladesh, thousands of species are known to have medicinal value and the use of different parts of several medicinal plants to cure specific ailments has been in vogue since ancient times. The beneficial medicinal effects of plant materials typically results from combination of secondary product present in plant. In plants these compounds are mostly secondary metabolites such as alkaloids, steroids, tannins, phenol compounds, resins, gums, flavonoids and fatty acids which are capable of producing definite physiological action on body (Joshi et al., 2009 ▶). 

*Premna serratifolia* Linn (Family: Verbenaceae) synonym of *Premna integrifolia*, otherwise known as “Agnimantha” in Ayurvedic system of medicine, is a small-sized tree or a large shrub, up to 9m in height with a comparatively short trunk and numerous branches. The plants grow wildly and planted in many districts of Bangladesh (Ghani, 2008 ▶) and almost all parts of this plant i.e. root, leaf and bark have tremendous medicinal value. The roots are used in the treatment of diabetes, inflammations, swellings, bronchitis, dyspepsia, liver disorders, piles, constipation and fever (Gokhani et al., 2005 ▶). It is widely used by the traditional practitioners as cardiotonic, antibiotic, anti-coagulant, stomachic, carminative, hepatoprotective, antitumor etc. (Vadivu et al., 2009 ▶). 

Previous pharmacological studies include reports of hypolipidemic (Khanna et al., 1991 ▶), anti-inflammatory (Barik et al., 1992 ▶), antidiabetic (Kar et al., 2003 ▶), CNS depressant (Quais et al., 2011 ▶; Baby et al., 2011 ▶), and antitumor activity (Vadivu et al., 2009 ▶). Previous phytochemical investigations have revealed the presence of several glycoside including iridoid glycosides and phenylethanoids like premnethanoside A and B, (Sudo et al., 1997 ▶) some xanthones (Wang and Xu, 2003 ▶), steroids and saponins (Gopalakrishnan and Rathi, 2003 ▶), flavonoids, triterpenoids and diterpenoids (including premnones A and C) in *Premna serratifolia* leaves (Chin et al., 2006 ▶). Investigations on *Premna serratifolia* flower buds have revealed the presence of volatile oil comprising mainly 1-octen-3-ol, (Z)-n-hexanol, 2-phenyl ethyl alcohol, (E,Z)-2,4-nonadienal and linalool (Teai et al., 1998 ▶).

 The roots have been found to contain the mixture of mono-, di-, tri- terpene hydrocarbons and oxygenated biological materials including 1H-Cycloprop[e]azulen-7-ol, decahydro-1, 2-Furancarboxaldehyde, 5-(hydroxymethyl), 2-Hydroxy-3-methylbenzaldehyde, 2s,6s-2,6,8,8-Tetramethyltricyclodecan-2-ol, Benzofuran, 2,3-dihydro, n-Hexadecanoic acid, 2-Propenoic acid (Singh et al., 2011 ▶). With a view to find the pharmacological rationale for some of the reports and traditional uses of the plant, the methanolic extract of *Premna serratifolia* bark was evaluated for CNS depressant, analgesic and anti-inflammatory activities. 

## Materials and Methods

**Plant materials and Extraction**

The fresh barks of *Premna integrifolia* plant were collected from Sunderbans, Bagerhat in the month of September, 2009 and identified by DR. M.A. Razzaque Shah PhD, Tissue Culture Specialist, BRAC Plant Biotechnology Laboratory, Bangladesh. The dried and coarsely powdered leaves (400 g) were extracted with methanol at room temperature for 72 h. The filtrate was evaporated to dryness under reduced pressure (45 ◦C) to afford the crude extract (yield ca. 6%) used in pharmacological screening.

**Animals**

Young Long-Evans rats of either sex weighing about 160-180 g were used for the experiments. The rats were purchased from the animal Research Branch of the International Centre for Diarrhoeal Disease and Research, Bangladesh (ICDDR,B). They were used for the evaluation of neuropharmacological, analgesic and anti-inflammatory activities. The animals were housed under standard laboratory conditions (relative humidity 55–65%, room temperature 23.0±2.0 ◦C and 12-h light:12-h dark cycle). The animals were fed with a standard diet and water *ad libitum*. In all animal experiments, the guidelines of the Animal Experimentation Ethics Committee, ICDDR, B were followed.

**Drugs and chemicals**

Acetic acid was obtained from Merck, Germany. Tween-80 was obtained from BDH Chemicals, UK. Formalin was purchased from CDH, India. Normal saline solution was purchased from Beximco Infusion Ltd., Bangladesh. Indomethacin, and Diazepam were obtained from Square Pharmaceuticals Ltd., Bangladesh. All chemicals used were of analytical reagent grade.

**Preliminary phytochemical analysis**

The crude extract of *Premna integrifolia* bark was subjected to a preliminary phytochemical screening for the presence of steroids, flavonoids, tannins and saponins (Harborne, 1984 ▶).

**Acute toxicity test**

Animals were divided into five groups (*n *= 6 per group) which were administered different doses of the crude extract (62.5, 125, 250, 500, 1000, 2000 and 4000 mg/kg p.o.), while the control group received only the vehicle (1% Tween 80 in water, p.o.). The general signs and symptoms of toxicity were observed for 24 h and mortality was recorded for each group at the end of this period (Lorke, 1983 ▶).

**CNS depressant activity**

*Hole cross test*

The method was done as described by Takagi et al. (1971) ▶. A steel partition was fixed in the middle of a cage (30 cm×20 cm×14 cm h). A hole (diameter 3 cm) was made in the steel partition at a height of 7.5 cm above the floor of the cage. The animals were divided into control, standard and test groups (*n *= 6 per group). The control group received vehicle (1% Tween 80 in water at the dose of 10 ml/kg p.o.) whereas the test group received the crude extract (at the doses of 250 and 500 mg/kg p.o.) and standard group received Diazepam at the dose of 1mg/kg body weight orally. Each animal was then placed on one side of the chamber and the number of passages of each animal through the hole from one chamber to the other was recorded for 3 min on 0, 30, 60, 90, 120, 180 and 240 min during the study period.

*Open field test*

This experiment was carried out as described by Gupta et al. (1971) ▶. The animals were divided into control, standard and test groups (*n*=6 per group). The control group received vehicle (1% Tween 80 in water at the dose of 10 ml/kg p.o.). The test group received the crude extract (at the doses of 250 and 500 mg/kg p.o.) and standard group received Diazepam at the dose of 1mg/kg body weight orally. The animals were placed on the floor of an open field (100 cm×100 cm×40 cm h) divided into a series of squares. The number of squares visited by each animal was counted for 3 min on 0, 30, 60, 90, 120, 180 and 240 min after treatment.

**Analgesic activity**

*Acetic acid induced writhing method*

The analgesic activity of the samples was studied using acetic acid-induced writhing model in rats. Test samples (at the doses of 100 and 200 mg/kg) and vehicle (1% Tween 80 in water) were administered orally to rats (*n*=6) 30 minutes prior to intraperitoneal administration of 0.7% v/v acetic acid solution (0.1ml/10g). The positive control group received Diclofenac-Na at the dose of 10 mg/kg p.o. After an interval of 5 min, the rats were observed for specific contraction of body referred to as ‘writhing’ for the next 10 min (Ahmed et al., 2006 ▶).

*Formalin test*

The antinociceptive activity of the drugs was determined using the formalin test described by Dubuission and Dennis (1977) ▶. Control group received 5% formalin. 20 µl of 5% formalin was injected into the dorsal surface of the right hind paw 60 min after administration of MEPI (100 and 200 mg/kg, p.o.) and Indomethacin (10 mg/kg, p.o.). The rats were observed for 30 min after the injection of formalin and the amount of time spent licking the injected hind paw was recorded. The first 5 min post formalin injection is referred to as the early phase and the period between 15 and 30 min as the late phase. The total time spent licking or biting the injured paw (pain behavior) was measured with a stop watch. 

**Anti-inflammatory activity**

*Carrageenan induced paw edema test in rats*

Long-Evan rats (100-120 g) of both sexes were divided into four groups with five animals each. The test groups received 100 and 200 mg/kg body weight, p.o. of the extract (MEPI). The reference group received Indomethacin (10 mg/kg body weight, p.o.) while the control group received 3 ml/kg body weight normal saline. After 1 h, 0.1 ml, 1% carrageenan suspension in normal saline was injected into the subplanatar tissue of the right hind paw. The paw volume was measured at 1, 2, 3 and 4 h after carrageenan injection using a micrometer screw gauge. The percentage inhibition of the inflammation was calculated from the formula: 

% inhibition = (1-D_t/_D_o_) x 100 

Where, D_o_ was the average inflammation (hind paw edema) of the control group of rats at a given time, D_t_ was the average inflammation of the drug treated (i.e., extract or reference Indomethacin) rats at the same time (Winter et al., 1962).

**Statistical analysis**

All data were expressed as mean±SEM. One-way ANOVA followed by Dunnett’s multiple comparison tests was used to analyze the data obtained from *in vivo *experiments. All statistical analyses were performed with Prism 4.0 (GraphPad software Inc., San Diego, CA). *P*<0.05 was considered to be significant. 

## Results

**Preliminary phytochemical analysis and acute toxicity**

Results of the preliminary phytochemical analysis carried out on the crude methanolic extract indicated the presence of sterols, flavonoids, tannins and saponins. No lethal effects were observed within 24 h after the administration of the extract at any of the doses used, even at the highest dose tested (4000 mg/kg). Therefore, the lethal dose (LD_50_) of the extract in rats could not be determined. 

**Neuropharmacological activity**

*Hole-cross test*

Results of the hole-cross test followed a similar trend to the ones observed in the open-field test. They were statistically significant for all dose levels and followed a dose-dependent response. The depressing effect was most intense during the second (60 min) and third (90 min) observation periods (Table 1).

**Open-field test**

In the open-field test, MEPI leaves extract exhibited a decrease in the movement of the test animals at all dose levels tested. The results were statistically significant for all doses and followed a dose-dependent response (Table 2).

**Analgesic activity**

*Acetic acid-induced writhing test*

Table 3 shows the effects of the extract on acetic acid-induced writhing in rats. The oral administration of both doses of MEPI

significantly (p<0.001) inhibited writhing response induced by acetic acid in a dose dependent manner.

*Formalin test*

MEPI (100 and 200 mg/kg, p.o.) significantly (p<0.001) suppressed the licking activity in either phase of the formalin-induced pain in rats in a dose dependant manner (Table 4). MEPI, at the dose of 200 mg/kg body weight, showed the more licking activity against both phases of formalin-induced pain than that of the standard drug Diclofenac Na.

**Anti-inflammatory activity**

*Carrageenan induced paw edema test*

**Figure 1:**
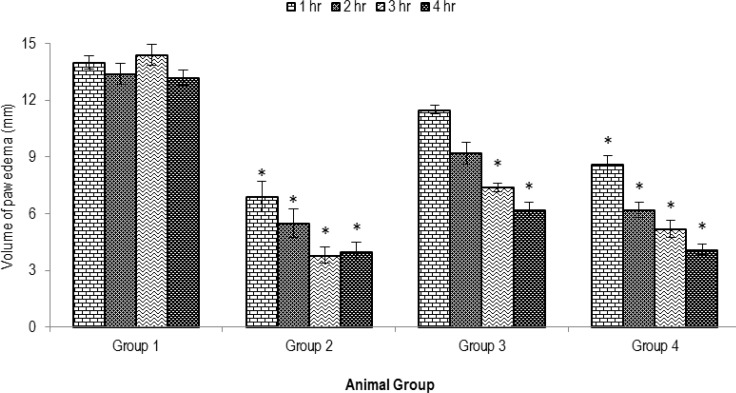
Effects of the methanolic extract of MEPI on carrageenan induced paw edema test. Values are mean±SEM, (n=5); *p<0.05 as compared to vehicle control (One way ANOVA followed by Dunnet test). Group I animals received vehicle (1% tween 80 in water), group II received indomethacin10 mg/kg body weight, group III, and IV, were treated with 100 and 200 mg/kg body weight (p.o.) of the MEPI

Figure 1 shows the results of the anti-edematous effects of orally administered of MEPI on carrageenan induced paw edema in rats. MEPI extract showed dose dependent anti-inflammatory activity and statistically significant (p<0.05). MEPI showed remarkable anti-inflammatory effects at 200 mg/kg dose (71.16% inhibition), whereas standard indomethacin showed 75.72% of inhibition of paw edema. 

**Table 1:** Effect of methanolic extract of *P. integrifolia* bark on hole cross test in rats

**Group**	**Dose**	**Number of Movements**				
		**0 min**	**30 min**	**60 min**	**90 min**	**120 min**
**Group-I**	10ml/kg,	118.4 ± 1.20	118 ± 1.30	115.4 ± 0.50	117.4 ± 1.16	118 ± 0.70
**Group-II**	1mg/kg,	117.2 ± 1.15	64.6± .43*	40.8± .58*	18.8± .86*	9.6 ± 0.50*
**Group-III**	100 mg/kg	118.4 ± 0.81	70.8±1.02*	51.8±1.35*	36.8± .02*	26 ± 0.71*
**Group-IV**	200 mg/kg	117.8 ± 1.43	66.8± .06*	43.6± .92*	26.6±0.92*	16.6± 0.60*

Values are mean±SEM, (n = 6);

* p<0.05, Dunnet test as compared to vehicle control. Group I animals received vehicle (1% tween 80 in water), group II received diazepam 1 mg/kg body weight, group III and group IV were treated with 100 and 200 mg/kg body weight (p.o.) of the MEPI.

**Table 2:** Effect of methanolic extract of *P. integrifolia* bark on Open Field test in rats

**Group**	**Dose**	**Number of Movements**				
		**0 min**	**30 min**	**60 min**	**90 min**	**120 min**
**Group-I **	10ml/kg	12.8 ± 1.15	13 ± 1.41	13.6 ± 0.92	14.2 ± 0.86	14 ± 0.54
**Group-II **	1mg/kg	11.2 ± 0.58	6 ± 0.70*	4 ± 0.83*	2.4±0.81*	1.8±0.37*
**Group-III **	100 mg/kg,	12 ± 0.70	7.8 ± 0.58*	5.4±0.50*	4.2±0.37*	3.2±0.37*
**Group-IV **	200 mg/kg,	12.2 ± 0.66	6.2 ± 0.37*	4 ± 0.70*	2.8±0.37*	1.4±0.40*

Values are mean±SEM, (n=6);

* p<0.05, Dunnet test as compared to vehicle control. Group I animals received vehicle (1% tween 80 in water), group II received diazepam 1 mg/kg body weight, group III and group IV were treated with 100 and 200 mg/kg body weight (p.o.) of the MEPI.

**Table 3:** Effect of methanolic extract of *P. integrifolia* bark on acetic acid induced writhing in rats

**Groups**	**Dose**	**No. of writhing**	**%inhibition**
**Group-I **	0.1 ml/10gm	26.33 ± 0.55	
**Group-II **	10mg/kg	10.83 ± 1.22*	58.8
**Group-III **	100mg/kg	13.0 ± 1.69*	50.63
**Group-IV **	200mg/kg	10.33 ± 0.76*	60.75

Values are mean±SEM, (n=5);

* p<0.05, Dunnet test as compared to vehicle control. Group I animals received vehicle (1% tween 80 in water), group II received diclofenac Na 10 mg/kg body weight, group III and group IV were treated with 100 and 200 mg/kg body weight (p.o.) of the MEPI.

**Table 4.** Effect of methanolic extract of *P. integrifolia* bark in hindpaw licking in the formalin test in rats

**Groups**	**Dose**	**Early phase (Sec)**	**% protection **	**Late phase (Sec)**	**% protection **
**Group-I **	10 ml/kg,	35.67 ± 1.38	-	46.0 ± 1.03	-
**Group-II **	10 mg/kg,	16.83 ± 0.90*	52.8	21.83 ± 0.70*	52.53
**Group-III **	100 mg/kg,	27.5 ± 0.76*	22.89	21.5 ± 0.95*	53.26
**Group-IV **	200mg/kg	16.17 ± 0.65*	54.67	18.0 ± 1.46*	60.86

Values are mean±SEM, (n=5);

* p<0.05, Dunnet test as compared to vehicle control. Group I animals received vehicle (1% tween 80 in water), group II received diclofenac Na 10 mg/kg body weight, group III and group IV were treated with 100 and 200 mg/kg body weight (p.o.) of the MEPI.

## Discussions

Locomotor activity is considered as an increase in alertness and decrease in locomotor activity indicating sedative effect (Verma et al., 2010 ▶). Extracts of *P. integrifolia* bark decreased locomotor activity indicates its CNS depressant activity. Gamma-aminobutyric acid (GABA) is the major inhibitory neurotransmitter in the central nervous system. Different anxiolytic, muscle relaxant, sedative-hypnotic drugs elucidate their action through GABA, therefore it is possible that extracts of MEPI may acts by potentiating GABAergic inhibition in the CNS via membrane hyperpolarization which leads to a decrease in the firing rate of critical neurons in the brain or may be due to direct activation of GABA receptor by the extracts (Kolawole et al., 2007 ▶). Many research showed that plant containing flavonoids, saponins and tannins are useful in many CNS disorders (Bhattacharya and Satyan, 1997 ▶). 

Earlier investigation on phytoconstituents and plants suggests that many flavonoids and neuroactive steroids were found to be ligands for the GABA_A_ receptors in CNS; which led to assume that they can act as Benzodiazepine like molecules (Verma et al., 2010 ▶). Phytochemical investigations also showed the presence of alkaloids, flavonoids, saponins and tannins in the extract, so might be these phytoconstituents are responsible for its CNS depressant activity. 

Acetic acid induced writhing response is a sensitive procedure to evaluate peripherally acting analgesics and represents pain sensation by triggering localized inflammatory response. Such pain stimulus leads to the release of free arachidonic acid from the tissue phospholipids (Ahmed et al., 2006 ▶). The response is thought to be mediated by peritoneal mast cells (Ribeiro et al., 2000 ▶), acid sensing ion channels (Voilley, 2004 ▶) and the prostaglandin pathways (Hossain et al., 2006 ▶). 

The organic acid has also been postulated to act indirectly by inducing the release of endogenous mediators, which stimulates the nociceptive neurons that are sensitive to NSAIDs and narcotics (Adzu et al., 2003 ▶). It is well known that non-steroidal anti-inflammatory and analgesic drugs mitigate the inflammatory pain by inhibiting the formation of pain mediators at the peripheral target sites where prostaglandins and bradykinin are proposed to play a significant role in the pain process (Hirose et al., 1984 ▶). In addition, it was suggested that non narcotic analgesics produce their action by interfering with the local reaction to peritoneal irritation thereby reducing the intensity of afferent nervous stimulation in the acetic acid induced writhing test, a model of visceral pain (Vogel and Vogel, 1997 ▶). 

The formalin model normally postulates the site and the mechanism of action of the analgesic (Chau, 1989 ▶). This biphasic model is represented by neurogenic (0-5 min) and inflammatory pain (15-30 min), respectively (Hunskaar and Hole, 1987 ▶). Drugs that act primarily on the central nervous system such as narcotics inhibit both as steroids and NSAIDs suppress mainly the late phase. The suppression of neurogenic and inflammatory pains by the extract might imply that it contains active analgesic principle that may be acting both centrally and peripherally. This is an indication that the extract can be used to manage acute as well as chronic pain. The mechanism by which formalin triggers C-fibers activation remained unknown for a relatively long time. 

Recently, however, McNamara et al. (2007) ▶ demonstrated that formalin activates primary afferent neurons through a specific and direct on TRPA1, a member of the transient receptor potential family of cation channels, expressed by a subset of C-fiber nociceptors and this effect is accompanied by increasing influx of Ca^2+^ ions. 

TRPA1 cation channels at primary sensory terminals were also reported to mediate noxious mechanical stimuli (Kerstein et al., 2009 ▶). These experiments suggest that Ca^2+^ mobilization through TRPA1cation channels is concomitant with noxious chemicals and mechanical stimuli as they produce their analgesic action. It is likely that the inhibitory effect of MEPI to pain response is due to inhibit the increase of the intracellular Ca^2+^ through TRPA1, presumably evoked by formalin. So, the bark extract of *P. intregifolia* may contain substances that affect the metabolism of Ca^2+^. Literature survey revealed that tannins, triterpenoids and flavonoid are the major phytoconstituents of* P. intregifolia* (Sudo et al., 1997 ▶; Chin et al., 2006 ▶). Flovonoids, for example, have been found to suppress the intracellular Ca^2+^ ion elevation in a dose dependent manner, as well as the release of pro-inflammatory mediators such as TNFα (Kempuraj et al., 2005 ▶). 

Carrageenan induced edema has been commonly used as an experimental animal model for acute inflammation and is believed to be biphasic. The early phase (1-2h) of the carrageenan model is mainly mediated by histamine, serotonin and increased synthesis of prostaglandins in the damaged tissue surroundings. The late phase is sustained by prostaglandin release and mediated by bradykinin, leukotrienes, polymorphonuclear cells and prostaglandins produced by tissue macrophages (Antonio and Brito, 1998 ▶; Gupta et al., 2006 ▶; Sawadogo et al., 2006). Since the extract significantly inhibits paw edema induced by carrageenan in the second phase and this finding suggests a possible inhibition of cyclooxygenase synthesis by the extract and this effect is similar to that produced by non-steroidal anti-inflammatory drugs such as Indomethacin, whose mechanism of action is inhibition of the cyclooxygenase enzyme. Flavonoids and saponins are well known for their ability to inhibit pain perception as well as anti-inflammatory properties due to their inhibitory effects on enzymes involved in the production of the chemical mediator of inflammation (Pin et al., 2010). 

Our preliminary pharmacological studies on the methanolic extract of *Premna integrifolia *bark provide in part scientific support for the use of this species in traditional medicine, particularly in various ailments related to CNS disorders, pain and inflammatory disseases. However, further pharmacological investigations are required to understand its underlying mode of action on the CNS, mechanism of pain inhibition and anti-inflammatory activities. In addition, future bioactivity-guided phytochemical work should be carried out to identify any active constituent(s).

## References

[B1] Adzu B, Amo S, Kapu SD, Gamaniel KS (2003). Anti-inflammatory and anti-nociceptive effects of Sphaeranthus senegalensis. J Ethnopharmacol.

[B2] Ahmed F, Hossain MH, Rahman AA, Shahid IZ (2006). Antinociceptive and sedative effects of the bark of Cerbera odollam Gaertn. J Ori Pharm Exp Med.

[B3] Antonio AM, Brito ARMS ( 1998). Oral anti-inflammatory and anti-ulcerogenic activities of a hydroalcoholic extract and partitioned fractions of Turnera ulmifolia (Turneraceae). J Ethnopharmacol.

[B4] Baby DA, Pothen N, Kurian DS, Jose J, james TS, Amal D (2011). Evaluation of anticonvulsant activity of Premna corymbosa in experimental mice. Int J Exp Pharmacol.

[B5] Barik BR, Bhowmik T, Dey AK, Patra A (1992). Premazole, an isoxazole alkaloid of Premna integrifolia and Gmelina arborea with anti-inflammatory activity. Fitoterapia.

[B6] Bauer AW, Kirby WMM, Sherris JC, Truck M (1966). Antibiotic susceptibility testing by a standardised single disk method. Am J Clin Pathol.

[B7] Bhattacharya SK, Satyan KS (1997). Experimental methods for evaluation of psychotropic agents in rodents: Anti-anxiety agents. Indian J Exp Biol.

[B8] Chau TT (1989).

[B9] Chin YW, Jones WP, Bachman I (2006). Cytotoxic clerodane diterpenoids from leaves of Premna tomentosa. Phytochem.

[B10] Dubuission D, Dennis SG (1977). The formalin test: A quantitative study of the analgesia effects of morphine, meperidine and brain stem stimulation in rats and cats. Pian.

[B11] Ghani A (2008). Medicinal Plants of Bangladesh: Chemical constituents and uses.

[B12] Gopalakrishnan S, Rathi BM (2003). Aerial parts of Premna tomentosa – An effective insecticide. National Seminar on New Millenium strategies for quality, safety and GMPS on herbal drugs or products. NBRI, Lucknow.

[B13] Gokhani RH, Lahiri SK, Santani DD, Shah MB (2005). Evaluation of immunomodulatory activity of Clerodendrum phlomidis and Premna integrifolia root. Int J Pharmacol.

[B14] Gupta BD, Dandiya PC, Gupta ML (1971). A psychopharmacological analysis of behavior in rat. Jpn J Pharmacol.

[B15] Harborne JB (1984). Phytochemical Methods (A Guide to Modern Techniques to Plant Analysis).

[B16] Hirose K, Jyoyama H, Kojima Y ( 1984). Pharmacological properties of 2-[44-(2-thiazolyloxy)-phenyl]-propionic acid (480156-5), a new non-steroidal anti-inflammatory agent. Arzneim ittelf Forsch. Drug Res.

[B17] Hossain MM, Ali MS, Saha A, Alimuzzaman M (2006). Antinociceptive activity of whole plant extracts of Paederia foetida. Dhaka Univ J Pharm Sci.

[B18] Hunskaar S, Hole K (1987). The formalin test in mice: Dissociation between inflammatory and non-inflammatory pain. Pain.

[B19] Joshi B, Lekhak S, Sharma A (2009). Antibacterial property of different medicinal plants: Ocimum sanctum, Cinnamomum zeylanicum, Xanthoxylum armatum and Origanum majorana. Kathmandu Uni J Sci Eng Tech.

[B20] Kar A, Choudhary BK, Bandyopadhyay NG (2003). Comparative evaluation of hypoglycaemic activity of some Indian medicinal plants in alloxan diabetic rats. J Ethnopharmacol.

[B21] Kempuraj D, Madhappan B, Kristodoulou S, Boucher W, Cao J, Papadopoulou N ( 2005). Flavonols inhibit proinflammatory mediators, intracellular calcium ion levels and protein kinase C theta phosphorylation in human mast cells. Br J Pharmacol.

[B22] Kerstein PC, Camino DD, Morgan MM, Stucky CL (2009). Pharmacological blockade of TRPA1 inhibits mechanical firing in nociceptors. Mol Pain.

[B23] Khanna AK, Chander R, Kapoor NK (1991). Hypolipidemic activity of Premna integrifolia in rats. Fitoterapia.

[B24] Kolawole OT, Makinde JM, Olajide OA (2007). Central nervous depressant activity of Russelia equisetiformis. Niger J Physiol Sci.

[B25] Lorke D (1983). A new approach to acute toxicity testing. Arch Toxicol.

[B26] McNamara CR, Mandel-Brehm J, Bautista DM, Siemens J, Deranian KL, Zhao M ( 2007). TRPA1 mediates formalin-induced pain. Proc Natl Acad Sci USA.

[B27] Qais N, Mahmud ZA, Karim MR, Bachar SC (2011). Anti-nociceptive, anti-inflammatory and sedative activities of leaf extracts of Premna exculenta (Roxb). J Pharm Res.

[B28] Rakh MS, Chaudhari SR (2010). Evaluation of CNS depressant activity of Momordica dioica Roxb willd fruit pulp. Int J Pharm Pharm Sci.

[B29] Rehka R (2010). Antimicrobial activity of different bark and wood of Premna serratifolia lin. Int J Pharm Bio Sci.

[B30] Ribeiro RA, Vale ML, Thomazzi SM, Paschoalato AB, Poole S, Ferreira SH, Cunha FQ (2000). Involvement of resident macrophages and mast cells in the writhing nociceptive response induced by zymosan and acetic acid in mice. Eur J Pharmacol.

[B31] Singh CR, Nelson R, Krishnan PM, Pargavi B (2011). Identification of volatile constituents from Premna serratifolia L through GC-MS. Int J Pharm Tech Res.

[B32] Sudo H, Takushi A, Ide T (1997). 10-o-acylated iridoid glucosides from leaves of Premna subscandens. Phytochem.

[B33] Takagi KM, Watanabe, Saito H (1971). Studies on the spontaneous movement of animals by the hole cross test: Effect of 2-dimethylaminoethane Its acylates on the central nervous system. Jpn J Pharmacol.

[B34] Teai T, Beanchini SP, Claude-Lafontaine A (1998). Volatile constituents of the flower buds of concrete of Premna serratofilia. J Essential Oil Res.

[B35] Vadivu R, Suresh JA, Girinath K, Kannan BP, Vimala R, Kumar NMS (2009). Evaluation of Hepatoprotective and In-vitro Cytotoxic Activity of Leaves of Premna serratifolia Linn. J Sci Res.

[B36] Verma A, Jana GK, Sen S, Chakraborty R, Sachan S, Mishra A (2010). Pharmacological Evaluation of Saraca indica Leaves for Central Nervous System Depressant Activity in Mice. J Pharm Sci Res.

[B37] Vogel HG, Vogel WH (1997). Drug Discovery and Evaluation – Pharmacological Assays.

[B38] Voilley N (2004). Acid-sensing ion channels (ASICs): New targets for the analgesic effects of Non-Steroid Anti-Inflammatory Drugs (NSAIDs). Curr Drug Targets Inflamm Allergy.

[B39] Wang DY, Xu SY (2003). Two new xanthones from Premna microphylla. Nat Pro Res.

